# Management of anticoagulant-related intracranial hemorrhage: an evidence-based review

**DOI:** 10.1186/cc13889

**Published:** 2014-05-23

**Authors:** Bappaditya Ray, Salah G Keyrouz

**Affiliations:** 1 Division of Critical Care Neurology, Department of Neurology, The University of Oklahoma Health Sciences Center, 920 Stanton L Young Blvd, Ste 2040, Oklahoma City, OK 73104, USA; 2 Department of Neurology, Washington University School of Medicine, 660 South Euclid Avenue, Box 8111, St Louis, MO 63110, USA

## Abstract

The increased use of anticoagulants for the prevention and treatment of thromboembolic diseases has led to a rising incidence of anticoagulant-related intracranial hemorrhage (AICH) in the aging western population. High mortality accompanies this form of hemorrhagic stroke, and significant and debilitating long-term consequences plague survivors. Although management guidelines for such hemorrhages are available for the older generation anticoagulants, they are still lacking for newer agents, which are becoming popular among physicians. Supportive care, including blood pressure control, and reversal of anticoagulation remain the cornerstone of acute management of AICH. Prothrombin complex concentrates are gaining popularity over fresh frozen plasma, and reversal agents for newer anticoagulation agents are being developed. Surgical interventions are options fraught with complications, and are decided on a case-by-case basis. Our current state of understanding of this condition and its management is insufficient. This deficit calls for more population-based studies and therapeutic trials to better evaluate risk factors for, and to prevent and treat AICH.

## Introduction

Use of anticoagulation therapy among the older western population for varied thromboembolic diseases potentially places them at risk of developing anticoagulant-related intracranial hemorrhage (AICH). The indications for use of anticoagulants are published in a guideline statement by the American College of Chest Physicians [1], to which readers are referred. AICH can be spontaneous or traumatic, and can occur in different intracranial compartments (for example, subdural hemorrhage, epidural hemorrhage, subarachnoid hemorrhage, or intracerebral hemorrhage (ICH)). Most clinical data on AICH are related to ICH, while extraparenchymal hemorrhages are reported but data on these are sparse.

**Figure 1:**
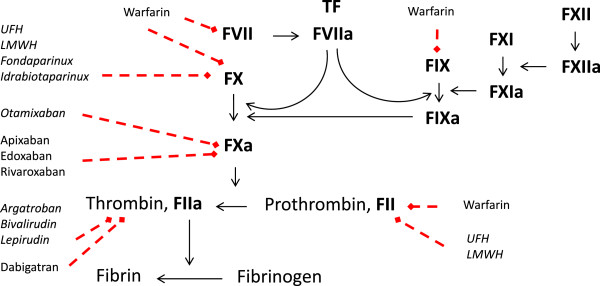
**Clinically available anticoagulants and their sites of action.** Dashed red arrows, sites of action. Italicized drugs are for parenteral use. F, factor; LMWH, low molecular weight heparin; TF, tissue factor; UFH, unfractionated heparin.

In the absence of clinical evidence, definitive guidelines, and proven therapies, clinicians are left scrambling for rapid correction of the coagulopathy and maintaining homeostasis to prevent secondary brain injury. The basic pharmacological properties of commonly used anticoagulants, their mechanism of action, and their indications are presented in Table 1 and Figure 1. Interested readers are referred to recently published articles for in-depth reviews of these agents [2,3]. The present review will primarily focus on the importance and impact of AICH, and, where available, the evidence-based management of this mostly iatrogenic disease.

**Table 1:** Pharmacologic properties of anticoagulants

**Drug**	**Target**	**Antithrombin dependent**	**Route**	**Half-life (hours)**	**Protein binding**	**Renal excretion**	**Monitoring**	**Antidote**	**Common indications**
Warfarin	Factors II, VII, IX, X; proteins C, S	No	p.o.	30 to 40	99%	92%	INR	Vitamin K	Thromboembolic prophylaxis in AF. Treatment of VTE. Thrombosis prophylaxis in prosthetic valve
UFH	Factors II, Xa (VIIa, IXa, XIa, XIIa)	Yes	i.v., s.c.	0.5 to 2.5 (dose dependent)	Variable	Mostly after hepatic metabolism	aPTT	Protamine sulfate	ACS. Thromboprophylaxis. Thromboembolic diseases (including ischemic stroke, CVST) in acute phase
LMWH	Factors IIa, Xa	Yes	s.c., i.v.	Variable according to the product	Variable	40% (10% unchanged)	Anti-factor Xa	Protamine sulfate (60%)	ACS. Thromboprophylaxis. Thromboembolic diseases
Fondaparinux	Factor Xa	Yes	s.c.	17 to 21	94%	~100% (77% unchanged)	Anti-factor Xa	None (see text)	VTE. Thromboprophylaxis. Selected cases of HIT
Argatroban	Factor IIa	No	i.v.	0.75 (prolonged in hepatic dysfunction)	54%	22% (16% unchanged)	aPTT, ACT	None	HIT. Thromboprophylaxis in patients suspected of HIT. ACS
Bivalirudin	Factor IIa	No	i.v.	0.5 (prolonged in renal impairment)	Only to factor IIa	20% unchanged	ECT (PT, aPTT, ACT has nonlinear prolongation)	None	HIT. ACS after thrombolysis. Thromboembolic prophylaxis during interventional procedures
Dabigatran	Factor IIa	No	p.o.	12 to 14	35%	80%	Modified TT/ECT/anti-factor IIa (also see Table 3)	PCC/FEIBA™/rFVIIa (see text)	Thromboembolic prophylaxis in AF. Treatment and thromboprophylaxis of VTE
Apixaban	Factor Xa	No	p.o.	8 to 14	87%	~25%	Anti-factor Xa (also see Table 3)	PCC/FEIBA™/rFVIIa (see text)	Thromboembolic prophylaxis in AF. Treatment and thromboprophylaxis of VTE
Rivaroxaban	Factor Xa	No	p.o.	7 to 11	93%	66% (33% unchanged)	Anti-factor Xa (also see Table 3)	PCC/FEIBA™/rFVIIa (see text)	Thromboembolic prophylaxis in AF. Treatment and thromboprophylaxis of VTE

ACS, acute coronary syndrome; ACT, activated clotting time; AF, atrial fibrillation; aPTT, activated partial thromboplastin time; CVST, cerebral venous sinus thrombosis; ECT, ecarin clotting time; HIT, heparin-induced thrombocytopenia; INR, International Normalized Ratio; i.v., intravenous; LMWH, low molecular weight heparin; PCC, prothrombin complex concentrate; p.o., per oral; PT, prothrombin time; rFVIIa, activated recombinant factor VII; s.c., subcutaneous; TT, thrombin time; UFH, unfractionated heparin; VTE, venous thromboembolism. FEIBA™ from Baxter (Deerfield, IL, USA).

## Epidemiology

The use of anticoagulants has increased exponentially over the last two decades along with the incidence of atrial fibrillation in aging western populations [4-6]; 5.6 million patients in the USA will be on anticoagulation for atrial fibrillation by 2050 [7]. With this increased use, the incidence of AICH has risen; the Greater Cincinnati area has seen a threefold increase in incidence during a 10-year period [8]. It is unclear whether a similar trend is still persistent with the introduction of newer generation oral anticoagulants (see below). Warfarin – the most commonly used, and therefore most extensively studied, long-term oral anticoagulant – is responsible for 9 to 14% of ICH [9-11] and carries 0.3 to 3.7% annual risk of warfarin-related intracerebral hemorrhage (WRICH) when the International Normalized Ratio (INR) is between 2 and 4.5 [12]. The risk of ICH in patients on long-term anticoagulation is 8 to 11 times higher than that of age-matched cohorts not taking anticoagulants [13,14]. Advanced age (≥70 years) [15,16], hypertension [9,14,15,17] and concurrent use of single or dual antiplatelets [18] are risk factors for WRICH. Other established risk factors include the early period after warfarin initiation [9,13,19], supratherapeutic levels of anticoagulation [9,14,17,20-23] and associated leukoaraiosis [21,23].

Similarly, unfractionated heparin (UFH) – a parenteral anticoagulant for various indications – also carries a risk of AICH. The incidence of symptomatic ICH used with UFH or heparinoids is 1 to 2.7% in patients with acute ischemic stroke [24,25]. A direct thrombin inhibitor, argatroban, another commonly used short-term parenteral anticoagulant, is associated with a 4.3% incidence of AICH [26].

In contrast, there are promising yet limited clinical data on AICH associated with the use of newer oral anticoagulants. The results of recent clinical trials using these drugs are presented in Table 2. The rates of ICH were 0.74%, 0.30%, and 0.23% per year among those assigned to warfarin, dabigatran 150 mg, and dabigatran 110 mg respectively in the RE-LY (Randomized Evaluation of Long Term Anticoagulant Therapy) trial, which assessed the efficacy of this oral direct thrombin inhibitor in atrial fibrillation [27]. In contrast, in the RE-COVER (Efficacy and Safety of Dabigatran Compared to Warfarin for 6 Month Treatment of Acute Symptomatic Venous Thromboembolism) trial, which compared dabigatran and warfarin in acute venous thromboembolism, there were no incidences of ICH during 6 months of therapy [28]. The yearly incidence of AICH ranged between 0.33 and 0.35% for apixaban, an oral factor Xa inhibitor, in two different trials – the ARISTOTLE (Apixaban for Reduction in Stroke and Other Thromboembolic Events) trial and the AVERROES (Apixaban Versus Acetylsalicylic Acid to Prevent Strokes) trial [29,30]. The incidence of AICH linked to rivaroxaban, another oral factor Xa inhibitor, in the ROCKET-AF (Rivaroxaban Once-daily Oral Direct Factor Xa Inhibition Compared with Vitamin K Antagonism for Prevention of Stroke and Embolism Trial in Atrial Fibrillation) study was 0.49%; that of warfarin was 0.74% (*P* < 0.05) [31]. However, in the EINSTEIN-PE (Oral Rivaroxaban Alone for the Treatment of Symptomatic Pulmonary Embolism) trial – a study that compared the efficacy of rivaroxaban with that of enoxaparin followed by warfarin in patients with pulmonary embolism – the incidence of AICH was lower for rivaroxaban (0.1% vs. 0.5%; *P* = 0.003) [32]. For cerebrovascular indication, national registry data showed similar peri-procedural incidence of ICH with bivalirudin and UFH after carotid stenting (0.1% vs. 0.2%; odds ratio = 0.62, 95% confidence interval = 0.20 to 1.91; *P* = 0.41) [33].

**Table 2:** Intracranial hemorrhage rates reported in phase 3 clinical trials of dabigatran, rivaroxaban and apixaban related to stroke prevention and treatment of acute venous thromboembolism

**Clinical trial**	**Indication**	**Inclusion criteria**	**Drug**	**Primary outcome**	**Intracranial bleeding**
RE-LY	Stroke prevention in nonvalvular atrial fibrillation	AF patients with moderate to high risk of stroke or systemic embolism with at least one of the following: age >75 years; h/o TIA or stroke; LVEF <40%; NYHA class II or higher; age 65 to 74 years with either DM, CAD, hypertension	Dabigatran 110 mg or 150 mg BID; warfarin with target INR 2 to 3	Prevention of stroke/systemic embolism: dabigatran 110 mg, 1.54%/year; dabigatran 150 mg, 1.11%/year; warfarin, 1.71%/year	AICH: dabigatran 110 mg, 0.23%/year; dabigatran 150 mg, 0.30%/year (*P* < 0.05); warfarin, 0.74%/year
ROCKET-AF	Stroke prevention in nonvalvular atrial fibrillation	AF with history of stroke or TIA. AF with two or more of the following: symptomatic heart failure or LVEF <35%; age >75 years; DM	Rivaroxaban 20 mg daily; warfarin with target INR 2 to 3	Prevention of stroke/systemic embolism: rivaroxaban, 2.12%/year; warfarin, 2.42%/year	AICH (all): rivaroxaban, 0.49%/year (*P* < 0.05); warfarin, 0.74%/year. ICH: rivaroxaban, 0.33%/year; warfarin, 0.49%/year. SDH: rivaroxaban, 0.13%/year; warfarin, 0.27%/year
ARISTOTLE	Stroke prevention in nonvalvular atrial fibrillation	AF with one or more of following: TIA or systemic embolism; symptomatic CHF or LVEF ≤40%; DM or hypertension on pharmacological treatment	Apixaban 5 mg BID; warfarin with target INR 2 to 3	Prevention of stroke/systemic embolism: apixaban, 1.27%/year; warfarin, 1.60%/year	AICH: apixaban, 0.33%/year; warfarin, 0.80%/year
RE-COVER	VTE recurrence prevention	Acute symptomatic DVT of legs or PE	Dabigatran 150 mg BID; warfarin with target INR 2 to 3	Thromboembolism or related deaths: dabigatran, 2.4%; warfarin, 2.1%	AICH: dabigatran, 0; warfarin, 0.24%
RECOVER II	VTE recurrence prevention	Acute symptomatic DVT of legs or PE	Dabigatran 150 mg BID; heparin/enoxaparin followed by warfarin with target INR 2 to 3	Recurrent VTE: dabigatran, 2.3%; warfarin, 2.2%	Not reported
EINSTEIN-DVT	Prevention of VTE recurrence	Acute symptomatic DVT	Rivaroxaban 15 mg BID × 3 weeks followed by 20 mg daily. Standard therapy: enoxaparin 1 mg/kg BID; bridging warfarin or acenocoumarol therapy with target INR 2 to 3	Recurrent VTE: rivaroxaban, 2.1%; enoxaparin-VKA, 3.0%	AICH data not reported separately. Bleeding in critical location: rivaroxaban, 0.2%; enoxaparin-VKA, 0.2%
EINSTEIN-PE	VTE prevention	Acute PE	Rivaroxaban 15 mg BID × 3 weeks followed by 20 mg daily. Standard therapy: enoxaparin 1 mg/kg BID; bridging warfarin or acenocoumarol therapy with target INR 2 to 3	Recurrent VTE: rivaroxaban, 2.1%; standard therapy, 1.8%	AICH: rivaroxaban, 0.12%; standard therapy, 0.50%

AF, atrial fibrillation; AICH, anticoagulant-related intracranial hemorrhage; ARISTOTLE, Apixaban for Reduction in Stroke and Other Thromboembolic Events; BID, twice daily; CAD, coronary artery disease; CHF, congestive heart failure; DM, diabetes mellitus; DVT, deep venous thrombosis; EINSTEIN-PE, Oral Rivaroxaban Alone for the Treatment of Symptomatic Pulmonary Embolism; h/o, history of; INR, International Normalized Ratio; LVEF, left ventricular ejection fraction; NYHA, New York Heart Association; PE, pulmonary embolism; RE-COVER, Efficacy and Safety of Dabigatran Compared to Warfarin for 6 Month Treatment of Acute Symptomatic Venous Thromboembolism; RE-LY, Randomized Evaluation of Long Term Anticoagulant Therapy; ROCKET-AF, Rivaroxaban Once-daily Oral Direct Factor Xa Inhibition Compared with Vitamin K Antagonism for Prevention of Stroke and Embolism Trial in Atrial Fibrillation; SDH, subdural hematoma; TIA, transient ischemic attack; VKA, vitamin K antagonist; VTE, venous thromboembolism.

Outcomes after AICH have been mostly studied in patients on warfarin. Patients with WRICH fare worse than those with ICH without coagulopathy, with case fatality rates ranging between 44 and 68% [34-36]. In the Greater Cincinnati study, patients on warfarin with INR > 3.0 had larger hematomas than other patients [37]. After presentation, hematoma enlargement is more common and could still occur beyond the first 24 hours in anticoagulated patients [37-39]. What is more worrisome is that hematoma could still expand despite rapid correction of coagulopathy [40]. Little is known about the outcome of patients with AICH related to the newer anticoagulants; however, with an expected rise in use as they get wider acceptance [41], physicians on the forefront are likely to encounter more patients with such hemorrhages.

## Management

There are several uncertainties in the management of AICH. These uncertainties include the lack of evidence and limited guidelines for using biological products and pharmacologic agents, and accurate, quick, and meaningful laboratory testing to evaluate the hemostatic system. Moreover, individual variation in response to therapy and the difficulty in assessing ongoing bleeding make it even more challenging to manage this clinical problem [42]. One should also note that the mere correction of a laboratory parameter (that is, an *ex vivo* test) might not correlate with reversal of coagulopathy *in vivo* as often demonstrated by the expansion of AICH even after such correction [40]. Given the emergent nature of AICH and their unpredictability, randomized clinical trials or even large cohort studies in this population are unlikely and recommendations regarding treatment strategies will continue to be based on case series and anecdotal experience [43].

## Principles and interpretation of monitoring anticoagulant therapy

Evaluating and monitoring blood coagulation parameters is imperative after AICH. This is particularly true when obtaining history is difficult, thus precluding knowledge of the culprit anticoagulant, information that is crucial to guide therapy. Although coagulation tests are mere surrogate markers for hemostasis, the effect of different anticoagulants on the coagulation system is important knowledge for the treating clinician to have. Some of these tests are quantitative, and others provide only qualitative information. Moreover, it is important to understand that testing techniques and their sensitivities vary widely especially with newer anticoagulants [44].

Routine and commonly used *ex vivo* coagulation tests are the prothrombin time (PT), INR, and activated partial thromboplastin time (aPTT); the thrombin time, ecarin clotting time, activated clotting time and endogenous thrombin potential are also available, albeit less widely. The plasma factors responsible for different coagulation assays and their alteration with oral anticoagulants are presented in Table 3[45,46]. In patients with ongoing hemorrhage, the PT is preferred over the aPTT for the estimation of coagulation factor levels, because the results are quickly available, it offers a good correlation with average factor concentrations and response to plasma replacement, and there is no interference with nonspecific lupus anticoagulant inhibitors, elevated factor VIII, and heparin contamination [47]. The modified thrombin time (also commercially known as the HEMOCLOT thrombin time assay; Aniara, West Chester, OH, USA) and ecarin clotting time are the best tests for measuring the anticoagulant effect of dabigatran [48]. A normal thrombin time would exclude clinically significant dabigatran in systemic circulation. Although anti-factor IIa level testing is available at present, not enough information on its characteristics is known – such as linearity for and responsiveness in patients on dabigatran. To the contrary, the anti-factor Xa level has good correlation with rivaroxaban/apixaban activity. The recommended test to measure the anticoagulant effect of rivaroxaban is the PT (using reagent Neoplastin Plus^®^; Diagnostica Stago, Asnières-sur-Seine, France) and anti-factor Xa assay. Dabigatran and rivaroxaban drug levels can be used as surrogate markers to assess the need for anticoagulation reversal, but they are also not widely available [48-50].

**Table 3:** Interpretation of coagulation tests with US Food and Drug Administration approved oral anticoagulants

**Coagulation test**	**Factors evaluated**	**Interpretation with dabigatran**	**Interpretation with apixaban**	**Interpretation with rivaroxaban**	**Interpretation with warfarin**
PT	Factors II, V, VII, X, fibrinogen	Linear and dose dependent prolongation; at therapeutic range prolongs 1.2 times the basal value	Prolonged but not well studied or standardized	Linear and dose dependent prolongation; at therapeutic range prolongs 1.5 times the basal value	Prolonged
aPTT	Factors II, V, VIII, IX, X, fibrinogen	Qualitative; prolongation dose dependent but not linear; at therapeutic range prolongs 2.5 times the basal value	Prolonged but not well studied or standardized	Linear and dose dependent prolongation; at therapeutic range prolongs 1.5 times the basal value; not as sensitive as PT	May be prolonged
TT	Fibrinogen	Linear and dose dependent prolongation; but prolongation may be excessive and requires dilution of the plasma samples	Data not available	Not affected	Not affected
ECT	Factor II	Linear and dose dependent prolongation; at therapeutic range prolongs three times the basal value	NA	NA	NA
ETP	Factor II	Decreased – concentration dependent	Decreased – concentration dependent	Decreased – concentration dependent	NA

aPTT, activated partial thromboplastin time; ECT, ecarin clotting time; ETP, endogenous thrombin potential (thrombin generation assay); NA, not applicable; PT, prothrombin time; TT, thrombin time.

Both warfarin and heparin have good linear correlation with the PT/INR and the aPTT respectively, but the utility of the traditional coagulation profile testing is questioned with the advent of newer anticoagulants (see Table 3). Viscoelastic assays that measure whole blood coagulation and provide a dynamic coagulation profile, such as thromboelastography and rotational thromboelastometry, are being increasingly used to provide rapid assessment of hemostasis [51]. These assays measure the increasing viscoelasticity of blood as it clots, which is proportional to clotting factors and platelet count/function [52]. Their advantages are a rapid turnaround time, and the detection of fibrinolysis. Clinical situations where viscoelastic assays have been used to evaluate hemostasis include trauma resuscitation [53], during cardiopulmonary bypass [54] and AICH [55]. One study reported dose-dependent shortening of the clot-lysis time in the presence of dabigatran [56]. These laboratory tests might hold promise in the near future, although further studies and experience are needed.

## Reversal of anticoagulation

Most authorities consider rapid and adequate reversal of anticoagulation as the cornerstone of therapy in AICH, despite the lack of evidence showing that correction of coagulopathy reduces the incidence of hematoma growth or improves outcome [40]. In certain situations, however – such as the presence of a mechanical valve, INR < 3, small hemorrhage, or no surgical intervention needed – some authorities advocate holding warfarin without actively reversing coagulopathy [57]. Antidotes specific to each anticoagulant are presented in Table 1. Although warfarin remains the most commonly used oral anticoagulant, newer agents are gaining popularity. The exact mechanism of the occurrence of WRICH remains speculative, but potentially involves a local vasculopathy and/or systemic factors (for example, higher prevalence of the apolipoprotein E ϵ2 allele in patients with WRICH) [11,58]. Preventative measures to mitigate WRICH could therefore be relatively inefficient, and the onus is on rapid coagulopathy reversal [59]. The same probably holds true for AICH related to newer anticoagulants. Hence, the first step in any AICH is to discontinue the offending drug, administer an antidote (if available) and monitor adequate reversal of anticoagulation. In the case of warfarin, theoretically adequate hemostasis is restored at an INR of ~1.5 since adequate quantities of the factors needed for *ex vivo* coagulation are restored. However, an optimal INR value is unknown. Unfortunately, parameters for adequate reversal with newer anticoagulants are yet to be defined. Despite concern among physicians for reversing anticoagulation in a prothrombotic state, available evidence with WRICH, albeit meager, supports correcting the coagulopathy in such cases [59-61].

### Vitamin K

Vitamin K 5 to 10 mg intravenously by slow infusion over 30 minutes is administered to all patients with WRICH. Anaphylactic reactions are uncommon, and are mitigated by a slow infusion rate. The subcutaneous route for the administration of vitamin K is less effective because of erratic absorption, and the enteral route provides slow absorption although it has an equivalent bioavailability to the intravenous route [62]. The action of vitamin K is slow; it takes 6 to 24 hours to replenish adequate concentrations of factors II, VII, IX and X and to restore hemostasis [63,64]. This warrants the administration of fresh frozen plasma (FFP), prothrombin complex concentrate (PCC) and/or recombinant activated factor VII (rFVIIa) to rapidly replenish deficient factors. One should note that these agents have a short half-life, and are practically used as bridging therapy until vitamin K becomes effective.

### Fresh frozen plasma

FFP contains all coagulation factors and is the most commonly used reversal agent in the USA due to its wide availability and physicians’ familiarity with it. The recommended dose is anywhere between 10 and 40 ml/kg [12,64]; in clinical practice, however, FFP is commonly under-dosed for fear of volume overload. Allergic reactions, possible transmission of infectious diseases, transfusion-related acute lung injury [65] and, most importantly, a time lag to administration (due to thawing, cross-matching, prolonged infusion time) have sparked interest in a more optimal way to replete factors. Lee and colleagues reviewed records of 45 patients with WRICH treated with FFP [66]. The median time from door to INR normalization was 30 hours (14 to 49.5 hours), with four patients’ hematomas enlarging after normalization. The disadvantages of using FFP can be mitigated by procurement of male donors to minimize transfusion-related acute lung injury or of universal (AB group) donors, and by using liquid plasma – plasma that is never frozen, thus bypassing the thawing process [67].

### Prothrombin complex concentrate

Commonly available PCCs contain varying combinations and concentrations of factor II, VII, IX and X, protein C, protein S and protein Z. Some contain heparin and antithrombin to reduce thrombogenic potency. PCCs are prepared from virally inactivated plasma, pooled from several donors [43]. The concentrates come in two forms: one containing the inactivated form of coagulation factors, and another containing the activated form, such as FEIBA™ (Baxter, Deerfield, IL, USA).

Inactivated four-factor PCCs are available in Europe and Australia. Until recently, only three-factor PCCs (factor II, IX and X) have been used in the USA. Kcentra™ (CSL Behring, King of Prussia, PA, USA) is the first inactivated four-factor PCC approved by the US Food and Drug Administration to correct warfarin-related coagulopathy. The lack of comparative data on the efficacy of the three-factor versus four-factor PCCs makes it difficult to recommend one over the other [43], but the theoretical disadvantage of three-factor PCC led clinicians to supplement it with either FFP or rFVIIa. In clinical practice, PCCs are dosed based on factor IX content in a given preparation. The half-lives of the component factors II, VII, IX, and X are 60 hours, 4 to 6 hours, 17 to 24 hours, and 31 hours, respectively [68]. The dose varies between 25 and 100 units/kg, depending on the degree of INR derangement, and is infused, following reconstitution, at a rate of 100 units/minute [69,70]. The efficacy and safety of four-factor PCC as compared with FFP were demonstrated in a recently reported randomized trial that showed faster repletion of coagulation factors and similar adverse effect profile in either cohort [71].

FEIBA™ is an anti-inhibitor coagulant complex that is US Food and Drug Administration approved for use in hemophiliacs. The complex has been used off-label in WRICH [72]. The recommended dose of FEIBA™ is 500 to 1,000 units, and the infusion rate should not exceed 2 units/kg/minute. The INR and fibrinogen should be checked 30 minutes after the end of infusion, and the dose may need to be repeated. Cryoprecipitate may be needed if the fibrinogen concentration is less than 100 mg/dl. Overcorrection of INR is possible and could lead to thrombotic complications such as superficial thrombophlebitis, arterial or venous thrombosis and disseminated intravascular coagulation. Such complications are mostly dose related and their incidence in warfarin reversal is low [59]. Moreover, these adversities could be partly due to the underlying primary disease process [71,73,74]. An important factor that limits the availability of PCCs is their prohibitive cost [69], although a recent analysis suggested that they might be more cost-effective than FFP for warfarin reversal after life-threatening hemorrhages such as ICH [75].

Studies have demonstrated rapid and effective reversal of the coagulopathy but PCC efficacy is still speculative in the setting of WRICH [76,77]. In a study of 55 patients with WRICH, 31 of whom were treated with PCC, there was faster INR correction and reduction of hematoma growth at 24 hours [76]. Conversely, in another study of 141 patients there was ICH expansion in 45.5% of patients, despite correction of coagulopathy; in-hospital mortality was 42.3% [40]. A retrospective study comparing FFP and PCC for the reversal of warfarin’s effect in WRICH did not demonstrate a significant difference in 30-day mortality [78]. An ongoing randomized control study is set to shed more light on this question [79]. In clinical practice, hemorrhages associated with the use of newer anticoagulants (dabigatran, rivaroxaban, and apixaban) are also managed with infusion of PCCs (25 to 50 units/kg) [80]. Both four-factor PCC and FEIBA™ (80 units/kg) have been demonstrated to increase thrombin generation in healthy volunteers on dabigatran and rivaroxaban [45,81].

### Recombinant activated factor VII

rFVIIa is a US Food and Drug Administration-approved treatment for congenital factor VII deficiency and hemophiliacs with inhibitors to factor VIII. This treatment has been used to correct coagulopathy associated with warfarin [82]. rFVIIa has a dual mechanism of action that involves initiating coagulation via the tissue factor and factor VIIa pathway and direct platelet activation resulting in a thrombin burst [83]. Given its short half-life (3 to 4 hours), INR reversal after rFVIIa dosing is transient, and vitamin K and PCC/FFP should be used concomitantly. Lower doses of 10 to 40 μg/kg are associated with a lower incidence of thrombotic complications [84,85]. Although INR is corrected rapidly using rFVIIa, it may simply reflect the *ex vivo* effect of factor VIIa on the PT assay and may not always correlate with clinical hemostasis [86,87]. This is also reflected in the *ex vivo* assays where rFVIIa has been shown to reverse INR in patients on warfarin with minimal effect on aPTT and bleeding time [88]. rFVIIa is effective in reversal of the anticoagulant effect of fondaparinux in healthy volunteers [89]. Its effectiveness, however, in fondaparinux-related ICH has not yet been demonstrated [80]. Administration of rFVIIa (120 μg/kg) in healthy volunteers on dabigatran and rivaroxaban did not correct thrombin generation tests [81]. The long-term clinical impact of rFVIIa in patients with AICH remains to be tested in prospective studies.

### Protamine

For symptomatic ICH with supratherapeutic aPTT associated with UFH, the infusion should be stopped and residual drug reversed using intravenous protamine sulfate at a ratio of 1 mg protamine per 100 units of UFH that was infused over the preceding 3 hours. Hemorrhagic conversion remains a threat up to 48 hours following reversal. Caution should be exercised by managing blood pressure [90], and the aPTT should be followed to monitor heparin neutralization. Side effects of protamine include hypotension, bradycardia, and anaphylactoid reactions. A paradoxical anticoagulant effect can be minimized by infusing protamine slowly, over 3 minutes, and avoiding doses greater than 50 mg at once. Protamine is less effective at neutralizing low molecular weight heparin (LMWH) because it only partially (60%) reverses its anti-factor Xa effect [80,91]. A longer half-life of subcutaneously administered LMWH further contributes to their sustained effects. Evidence-based guidelines on LMWH reversal is lacking. Following the manufacturer’s recommendations, 1 mg protamine should be administered for every 1 mg enoxaparin (Sanofi-Aventis, Bridgewater, NJ, USA), and 1 mg protamine for every 100 anti-Xa IU dalteparin (Eisai Co. Ltd, Tokyo, Japan) or tinzaparin (LEO Pharmaceutical Products, Ballerup, Denmark) that was administered in the preceding 8 hours. If bleeding persists, an additional half-dose of protamine is recommended [80,91].

### Antifibrinolytics

Tranexamic acid is an antifibrinolytic agent used for prevention of re-rupture of cerebral aneurysm after subarachnoid hemorrhage. Long-term use of tranexamic acid has been associated with hydrocephalus, thrombosis and delayed cerebral ischemia in this patient population [92]. Concomitant blood pressure reduction and rapid infusion of tranexamic acid has been shown to be associated with less frequent hematoma expansion in spontaneous ICH not related to anticoagulant use [93]. Animal data on hemostatic therapy with tranexamic acid in WRICH demonstrated lower efficacy as compared with PCC and rFVIIa and worsening perihematomal edema [94]. Since no antidote is currently available for clinical use in dabigatran-related major bleeding, some authorities recommend administration of 1 g tranexamic acid [95]. In addition, there is anecdotal experience of its use in a Jehovah’s Witness patient after post-tissue plasminogen activator-related ICH to prevent hemorrhage expansion [96].

### Special considerations with newer oral anticoagulants

To date, there are no specific antidotes available for clinical use to reverse the anticoagulant effect of newer oral agents such as dabigatran, apixaban, and rivaroxaban. Candidate antidotes for dabigatran and factor Xa inhibitors have demonstrated effectiveness in preclinical studies [97,98]. The proposed antidote for dabigatran is the antibody aDabi-Fab that achieves an affinity for dabigatran 350 times stronger than its affinity for thrombin. aDabi-Fab supposedly has no prothrombotic effect [98]. The antidote for factor Xa inhibitors could have wider applicability in reversing both oral and parenteral agents, including LMWH and fondaparinux. This antidote (PRT064445) reversed the inhibition of factor Xa by direct factor Xa inhibitors and corrected the prolongation of *ex vivo* clotting times in a dose-dependent fashion. PRT064445 restored hemostasis deranged by rivaroxaban in a rabbit model of liver laceration [97]. In rats, the antidote dose-dependently and completely corrected the increased blood loss caused by enoxaparin and fondaparinux [97].

Until specific antidotes are available for clinical use, the goal of treatment for AICH related to newer anticoagulants is to restore coagulation by thrombin generation and to overwhelm the effects of these agents with nonactivated four-factor PCC, FEIBA™ or rFVIIa [99,100]. Other measures may include prevention of absorption by administering activated charcoal for recent ingestion and hemodialysis or hemoperfusion [99]. The efficacy of prompt administration of activated charcoal has been demonstrated for dabigatran with *in vitro* models [99] and for rivaroxaban and apixaban with *in vivo* animal models [101,102]. In patients taking dabigatran and undergoing hemodialysis, there was an 85% reduction in plasma level at 60 minutes with charcoal hemoperfusion [103,104]. Rivaroxaban is unlikely to be dialyzable due to its high protein binding, although it may theoretically be eliminated via plasma exchange. In emergency situations such as AICH, however, neither hemodialysis nor plasma exchange is practically feasible.

## Other supportive measures

The general management of patients with AICH is outlined in recent guidelines published by the American Heart Association [105]. Here, we elaborate on the acute management of hypertension and surgical interventions, mostly relevant to intra-parenchymal bleed hemorrhages.

### Anti-hypertensive therapy

In the acute phase of ICH, elevated blood pressure is commonly encountered, with evidence linking acute hypertension to increased mortality, disability, and possibly risk of hematoma growth [106,107]. One argument against aggressive treatment of acute hypertension in ICH, reducing perfusion around the hematoma, was counteracted by evidence from positron emission tomography studies [108,109]. These studies and a handful of randomized controlled trials, most notably the INTERACT-2 (Intensive Blood Pressure Reduction in Acute Cerebral Hemorrhage Trial – 2) study, showed that aggressive blood pressure lowering in ICH to treat systolic blood pressure ≤140 mmHg is safe [110]. Although a difference in primary outcome was absent, there was improved morbidity among patients undergoing aggressive systolic blood pressure control. Results of an ongoing similar trial, the ATACH-II (Antihypertensive Treatment of Acute Cerebral Hemorrhage, Phase III) trial, are awaited and could add to our knowledge [111]. There remains a lack of consensus on how to manage acutely elevated blood pressure in patients with AICH since this population is excluded from acute ICH trials. However, extrapolating results from spontaneous ICH trials seems reasonable – although until such AICH trials are conducted, clinicians should use clinical judgment and manage acute hypertension on a case-by-case basis.

### Surgery

The rationale to evacuate cerebral parenchymal hemorrhages is to prevent thrombin and hemoglobin degradation-product-mediated secondary brain damage. In addition, surgical removal of blood clots alleviates pressure gradients inside the skull and restitutes normal anatomy, thus reducing or abolishing pressure on and injury to vital midline diencephalic structures. There are no studies specifically addressing the question of surgical evacuation of hematoma in AICH, whether it is intra-parenchymal or elsewhere. In clinical practice, and for obvious reasons, reversal of coagulopathy precedes potential hematoma evacuation.

Randomized trials for spontaneous ICH go back to the early 1960s. The STICH trial (International Surgical Trial in Intracerebral Hemorrhage) randomized over 1,000 patients to early surgery (within 24 hours of randomization) or initial conservative treatment, but failed to show any clinical benefit of surgical evacuation [112]. Subgroup analysis indicating potential benefit of surgery in superficial ICH (<1 cm from cerebral surface) was the basis for the recently concluded STICH II trial, in which primary intention-to-treat analysis showed a small but nonsignificant increase in the number of patients having a favorable outcome at 6 months in the early surgery group. There was also a suggestion of a reduction in mortality, but this finding was also nonsignificant [113].

Surgical management after traumatic brain injury leading to acute epidural or subdural hemorrhage is generally indicated if the Glasgow Coma Scale score is ≤8 and there is a 5 mm or more shift on head computed tomography [114,115]. In a retrospective case series of 11 patients on oral phenprocoumon (vitamin K antagonist) presenting with acute subdural hemorrhage, the authors reported mortality of 45.5% at 6 months and the remaining patients were functionally independent during the same follow-up period [116]. Given the acuity and associated high morbidity and mortality of AICH, it is difficult to systematically study surgical therapy in this subgroup of patients.

## Resumption of anticoagulation

Whether and when to resume anticoagulation are other important questions that haunt clinicians managing AICH. Again there is no straightforward and definitive answer to this dilemma. Very few data are available to guide clinicians on this important issue, and most of what we know is generated by small case series. The decision of whether to restart anticoagulation should take into consideration the recent anticoagulant-related hemorrhage and the need for anticoagulation. The evidence that supports use of anticoagulation in the disease process should be considered; and the urgency with which anticoagulation needs to be restarted and the risk of trauma/falls leading to rebleeding should also be a factor. The presence of microbleeds on brain magnetic resonance imaging might portend a higher risk of developing recurrent AICH once anticoagulation is restarted [117]. Finally, the availability of alternative approaches, *in lieu* of anticoagulation, to prevent progression of or complications from a thromboembolic disease process should be sought (for example, newer anticoagulants, temporary vena cava filter devices).

Claassen and colleagues reviewed medical records of 52 consecutive patients with WRICH [118]. Warfarin was restarted after a median of 10 days (range 7 to 28 days) in 23 patients; one-half of these had mechanical heart valves. During follow-up there were five cases of recurrent major bleeding in the group on warfarin, and five thromboembolic events in those not restarted on warfarin. In another retrospective study, Majeed and colleagues identified 234 patients with WRICH, 177 of whom had long-term follow-up [119]. Of these 177 patients, 59 resumed warfarin after a median of 5.6 weeks. Recurrent ICH occurred in eight patients who restarted warfarin and in 10 patients who did not. The combined risk of recurrent WRICH or ischemic stroke reached its nadir when warfarin was resumed 10 to 30 weeks after the incident WRICH.

In patients with a mechanical valve, the annual risk of systemic embolism is 4% per year, and the risk of valve thrombosis is 1.8% [59,64,120]. Given the risk of hematoma expansion and subsequent high morbidity associated with WRICH, it is advisable to withhold anticoagulation in the acute phase [59]. In the 2010 version of the American Heart Association guidelines, a specific mention of time to restart anticoagulation after WRICH was omitted [105]. The European stroke initiative suggests balancing the risk of recurrent ICH and thromboembolism in individuals with a strong indication for anticoagulation, and reinitiating the latter 10 to 14 days after the incident WRICH [64]. Practically, resumption of anticoagulation is debated among stroke experts and most agree to restart anticoagulation in 7 to 14 days provided benefits outweigh the risk and if the hemorrhage was deep in location [121].

## Conclusion

Many questions pertaining to the management of AICH remain unanswered. Conducting a systematic randomized trial in this subpopulation of patients is difficult. The available literature (although mostly retrospective and case series) recommends rapid correction of coagulopathy, early surgical intervention if appropriate and holding resumption of anticoagulation during the acute phase. Newer oral anticoagulants are marketed to be safe with lower incidence of AICH and can be potentially considered when resumption of anticoagulation is warranted. However, the lack of optimal reversal agent in case of complications may deter clinicians from using them. The development of safe and novel reversal agents for newer generation anticoagulants should thus take precedence, given their booming use and the current naivety and inexperience of the healthcare provider in managing their hemorrhagic complications. Better studies are needed to guide clinicians in appropriate management and to help prognostication. Given the overall poor functional outcome of patients with ICH in general, and perhaps AICH in particular, a need exists to explore preventative avenues. Hence, emphasis should be also placed on patient education, lifestyle and risk modification and discussion of the risk–benefit ratio before anticoagulants are prescribed.

## Abbreviations

AICH: Anticoagulant-related intracranial hemorrhage; aPTT: Activated partial thromboplastin time; FFP: Fresh frozen plasma; ICH: Intracerebral hemorrhage; INR: International Normalized Ratio; LMWH: Low molecular weight heparin; PCC: Prothrombin complex concentrate; PT: Prothrombin time; rFVIIa: Activated recombinant factor VII; UFH: Unfractionated heparin; WRICH: Warfarin-related intracerebral hemorrhage.

## Competing interests

The authors declare that they have no competing interests.
